# Robustness of Serologic Investigations for Chikungunya and Mayaro Viruses following Coemergence

**DOI:** 10.1128/mSphere.00915-19

**Published:** 2020-02-05

**Authors:** Carlo Fischer, Fernando Bozza, Xiomara Jeanleny Merino Merino, Celia Pedroso, Edmilson F. de Oliveira Filho, Andrés Moreira-Soto, Alvaro Schwalb, Xavier de Lamballerie, Eduardo Martins Netto, Patrícia T. Bozza, Manoel Sarno, Carlos Brites, Eduardo Gotuzzo, Michael Talledo, Jan Felix Drexler

**Affiliations:** a Charité-Universitätsmedizin Berlin, corporate member of Freie Universität Berlin, Humbolt-Universität zu Berlin and Berlin Institute of Health, Institute of Virology, Berlin, Germany; b Evandro Chagas National Institute of Infectious Disease, Oswaldo Cruz Foundation (FIOCRUZ), Rio de Janeiro, Brazil; c Instituto de Medicina Tropical Alexander von Humboldt, Universidad Peruana Cayetano Heredia, Lima, Peru; d Departamento de Medicina, Facultad de Medicina, Universidad Peruana Cayetano Heredia, Lima, Peru; e Complexo Hospitalar Universitário Professor Edgard Santos, Universidade Federal de Bahia, Salvador, Brazil; f Unité des Virus Émergents, Aix-Marseille University, IRD 190, INSERM 1207, IHU Méditerranée Infection, Marseille, France; g German Centre for Infection Research (DZIF)‡; h Martsinovsky Institute of Medical Parasitology, Tropical and Vector-Borne Diseases, Sechenov University, Moscow, Russia; University of Texas Southwestern Medical Center

**Keywords:** cross-reactivity, arbovirus diagnostics, serology, Brazil, Peru, ELISA, mosquito-borne disease, outbreak

## Abstract

Since 2013, the arthropod-borne Chikungunya virus (CHIKV) has cocirculated with the autochthonous Mayaro virus (MAYV) in Latin America. Both belong to the same alphavirus serocomplex, termed the Semliki Forest serocomplex. The extent of antibody cross-reactivity due to the antigenic relatedness of CHIKV and MAYV in commonly used serologic tests remains unclear. By testing 64 CHIKV- and 37 MAYV-specific sera from cohort studies conducted in Peru and Brazil, we demonstrate about 50% false-positive test results using commercially available enzyme-linked immunosorbent assays (ELISAs) based on structural antigens. In contrast, combining ELISAs for CHIKV and MAYV significantly increased positive predictive values (PPV) among all cohorts from 35.3% to 88.2% for IgM and from 61.3% to 96.8% for IgG (*P* < 0.0001). Testing of longitudinally collected CHIKV-specific patient sera indicated that ELISA specificity is highest for IgM testing at 5 to 9 days post-onset of symptoms (dpo) and for IgG testing at 10 to 14 dpo. IgG cross-reactivity in ELISA was asymmetric, occurring in 57.9% of MAYV-specific sera compared to 29.5% of CHIKV-specific sera. Parallel plaque reduction neutralization testing (PRNT) for CHIKV and MAYV increased the PPV from 80.0% to 100% (*P* = 0.0053). However, labor-intense procedures and delayed seroconversion limit PRNT for patient diagnostics. In sum, individual testing for CHIKV or MAYV only is prone to misclassifications that dramatically impact patient diagnostics and sero-epidemiologic investigation. Parallel ELISAs for both CHIKV and MAYV provide an easy and efficient solution to differentiate CHIKV from MAYV infections. This approach may provide a template globally for settings in which alphavirus coemergence imposes similar problems.

**IMPORTANCE** Geographically overlapping transmission of Chikungunya virus (CHIKV) and Mayaro virus (MAYV) in Latin America challenges serologic diagnostics and epidemiologic surveillance, as antibodies against the antigenically related viruses can be cross-reactive, potentially causing false-positive test results. We examined whether widely used ELISAs and plaque reduction neutralization testing allow specific antibody detection in the scenario of CHIKV and MAYV coemergence. For this purpose, we used 37 patient-derived MAYV-specific sera from Peru and 64 patient-derived CHIKV-specific sera from Brazil, including longitudinally collected samples. Extensive testing of those samples revealed strong antibody cross-reactivity in ELISAs, particularly for IgM, which is commonly used for patient diagnostics. Cross-neutralization was also observed, albeit at lower frequencies. Parallel testing for both viruses and comparison of ELISA reactivities and neutralizing antibody titers significantly increased diagnostic specificity. Our data provide a convenient and practicable solution to ensure robust differentiation of CHIKV- and MAYV-specific antibodies.

## Abstract (precis)

Geographically overlapping transmission of Chikungunya virus (CHIKV) and Mayaro virus (MAYV) in Latin America challenges serologic diagnostics and epidemiologic surveillance, as antibodies against the antigenically related viruses can be cross-reactive, potentially causing false-positive test results. We examined whether widely used ELISAs and plaque reduction neutralization testing allow specific antibody detection in the scenario of CHIKV and MAYV coemergence. For this purpose, we used 37 patient-derived MAYV-specific sera from Peru and 64 patient-derived CHIKV-specific sera from Brazil, including longitudinally collected samples. Extensive testing of those samples revealed strong antibody cross-reactivity in ELISAs, particularly for IgM, which is commonly used for patient diagnostics. Cross-neutralization was also observed, albeit at lower frequencies. Parallel testing for both viruses and comparison of ELISA reactivities and neutralizing antibody titers significantly increased diagnostic specificity. Our data provide a convenient and practicable solution to ensure robust differentiation of CHIKV- and MAYV-specific antibodies.

## OBSERVATION

**FIG 1:**
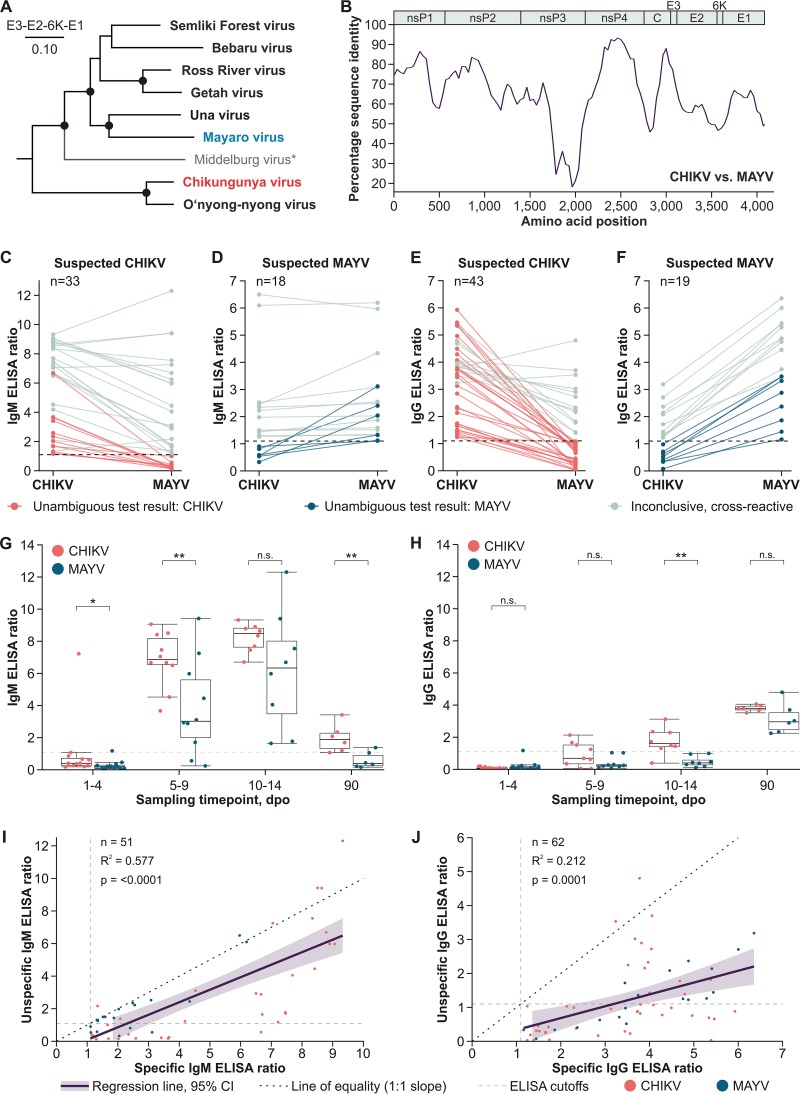
Phylogeny, antibody kinetics, and ELISA cross-reactivities of CHIKV and MAYV. (A) Maximum likelihood phylogeny of members of the Semliki Forest serocomplex based on translated amino acid sequences of the envelope and 6K protein-coding domains. A Whelan and Goldman substitution model was used in MEGA-X (https://www.megasoftware.net), with a discrete gamma distribution of site-specific rates and a complete deletion option. Statistical support of grouping was determined by 500 bootstrap replicates. For all viruses, the ICTV reference sequences were used (https://talk.ictvonline.org/ictv-reports/ictv_online_report/positive-sense-rna-viruses/w/togaviridae/872/genus-alphavirus). *, Middelburg virus was included to show the complete phylogeny, although it likely forms a distinct serocomplex. (B) Percentage amino acid sequence identity between CHIKV and MAYV calculated using the ICTV reference sequences and SSE version 1.3 (http://www.virus-evolution.org/Downloads/Software/), with a fragment length of 400 and an increment between fragments of 100 amino acid residues. (C) CHIKV and MAYV IgM ELISA reactivities in Brazilian CHIKV-specific sera. (D) CHIKV and MAYV IgM ELISA reactivities in Peruvian MAYV-specific sera. (E) CHIKV and MAYV IgG ELISA reactivities in Brazilian CHIKV-specific sera. (F) CHIKV and MAYV IgG ELISA reactivities in Peruvian MAYV-specific sera. (G) Median CHIKV and MAYV IgM ELISA reactivities of longitudinally sampled CHIKV-specific sera. *, *P* < 0.05; ** *P* < 0.01; n.s., differences were not significant. (H) Median CHIKV and MAYV IgG ELISA reactivities of longitudinal CHIKV-specific sera over time. (I) Linear regression of specific and unspecific CHIKV and MAYV IgM ELISA reactivities. 95% CI, 95% confidence interval. (J) Linear regression of specific and unspecific CHIKV and MAYV IgG ELISA reactivities. All nonlongitudinal samples were classified based on serologic test results. Conducted ELISAs are based on comparable recombinant structural proteins and CE (Conformité Européenne) labeled. For each ELISA, 1 μl patient serum was diluted 1:101 with sample buffer and applied to antigen-covered test wells. Human antibodies bound to the antigens were next targeted by peroxidase-labeled anti-human secondary antibodies. Afterwards, a substrate solution was added. The substrate was oxidized if peroxidase-labeled anti-human secondary antibodies were present, increasing the absorbance of the substrate solution. Absorbance was measured at 450 nm wavelength. Ratios were calculated using a calibrator sample provided in the kits.

Since 1955, Mayaro virus (MAYV) infections have been reported in Latin America, predominantly from the Amazon Basin (1, 2). In recent years, MAYV emergence in areas of previous nonendemicity has been observed (2, 3). Around 2013, Chikungunya virus (CHIKV) emerged in the Americas, infecting millions of individuals as of today (4). CHIKV and MAYV are both alphaviruses belonging to the Semliki Forest serocomplex (Fig. 1A), in which antibody cross-recognition of heterologous antigens can occur due to relatively high translated sequence identity between the protein-coding genomic domains (Fig. 1B) (5). As alphavirus viremia is short-lived, serologic detection of virus-specific antibodies is required for patient diagnostics and sero-epidemiologic studies (6, 7). Diagnostics in public health laboratories demand robust high-throughput tests, such as enzyme-linked immunosorbent assays (ELISAs) (7). To systematically assess serologic testing of MAYV and CHIKV, we assembled a panel comprising 37 MAYV-specific sera from Peru and 64 CHIKV-specific sera from Brazil (8), including longitudinally collected samples (6) (Table 1). Samples were tested using ELISA kits relying on comparable structural antigens that are widely used in Latin America (Euroimmun, Luebeck, Germany) (9, 10).

**TABLE 1:** Sample characteristics^*a*^

Region(s) of cohort site (country)	Study type	Study purpose	Yr(s)	No. of samples	Mean age (yr) of subjects (CI)	% female/ % male	Reference	Classification of sera
Bahia (Brazil)	Cross-sectional	Zika virus surveillance	2015–2016	28	41 (34, 47)	54/46	8	CHIKV
Rio de Janeiro (Brazil)	Prospective, longitudinal	Zika virus/CHIKV antibody kinetics	2016	36	41 (29, 53)	33/67	6	CHIKV
Loreto, Piura, Lambayeque (Peru)	Prospective	Malaria surveillance	2018	21	38 (28, 47)	55/45	NA	MAYV
Junín, Cusco, Loreto (Peru)	Prospective	Malaria surveillance	2001–2004	16	17 (10, 25)	50/50	NA	MAYV

a CI, 95% confidence interval; NA, not available; CHIKV, Chikungunya virus; MAYV, Mayaro virus. Sampling and testing were conducted in accordance with IRB approval numbers 1.408.499 and UPCH104562 and CAAE approval number 58782016.8.1001.5249.

Alphavirus-specific IgM detection is important for patient diagnostics and incidence estimates during surveillance (6, 11). Among IgM-positive sera in this study, 64.7% were ELISA positive for both CHIKV and MAYV. Cross-reactivities were comparable for CHIKV-specific (63.6%) (Fig. 1C) and MAYV-specific (66.7%) (Fig. 1D) sera. Detection of IgG is key for testing of convalescent-phase sera and in epidemiologic studies. Compared to that of IgM-positive sera, cross-reactivity was significantly lower among IgG-positive sera, of which 38.1% were ELISA positive for both viruses (*P* = 0.0081, Fisher’s exact test). IgG cross-reactivity in ELISA was not symmetric. A total of 29.5% of the CHIKV-specific (Fig. 1E) and 57.9% of the MAYV-specific sera (Fig. 1F) yielded positive test results for both viruses (*P* = 0.0484, Fisher’s exact test). An individual ELISA for CHIKV- or MAYV-specific antibodies, particularly for IgM, is thus not reliable in regions with CHIKV and MAYV cocirculation, as high rates of false-positive results must be expected.

Antibody maturation over time may affect the level of cross-reactivity in serologic tests (12). To examine how antibody cross-reactivity changes over time, longitudinally collected CHIKV-specific sera were tested. Using ELISA, CHIKV IgM reactivity increased rapidly, with a peak at 10 to 14 days post-onset of symptoms (dpo) (Fig. 1G). CHIKV IgG reactivity increased steadily, with 100% seroconversion at 90 dpo (Fig. 1H) (6). Contrarily to what was expected, decreasing cross-reactivity over time as a consequence of antibody maturation was not observed. Although MAYV ELISA reactivity was lower than that for CHIKV, the trends of CHIKV-specific ELISA reactivity and cross-reactive MAYV ELISA reactivity were identical for both IgM and IgG, suggesting that cross-reactivity was correlated with CHIKV-specific ELISA reactivity. To examine this potential correlation, we performed linear regression analyses. For both CHIKV- and MAYV-specific sera, IgM ELISA cross-reactivity correlated significantly with virus-specific IgM ELISA reactivity (*P* < 0.0001), explaining 57.7% of the observed variance (Fig. 1I). Similarly, IgG ELISA cross-reactivity correlated with virus-specific ELISA reactivity (*P* = 0.0001), explaining 21.2% of the observed variance (Fig. 1J). The risk of false-positive ELISA results, particularly for IgM, thus increases with increasing virus-specific ELISA reactivity.

**FIG 2:**
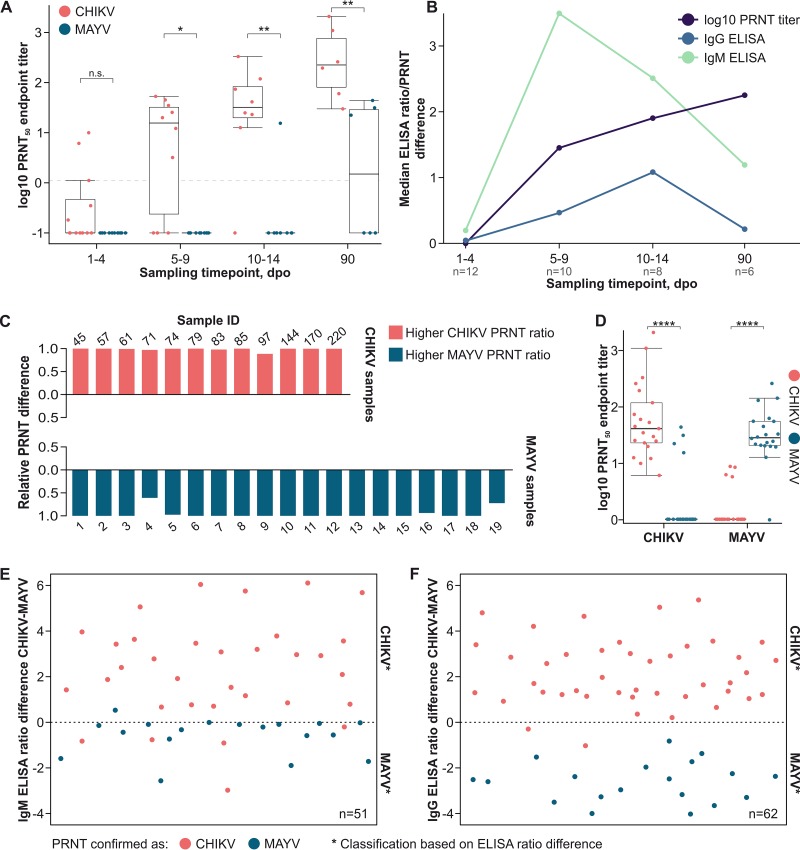
Serologic differentiation between CHIKV and MAYV. (A) Median CHIKV and MAYV PRNT endpoint titers of CHIKV-specific sera over time. Neutralizing antibody titers were calculated using the built-in variable slope model in GraphPad Prism 6 (GraphPad Software, LLC; https://www.graphpad.com). Statistical significance levels in panels A and D were determined by the Mann-Whitney U test. n.s., not significant; *, *P* < 0.05; **, *P* < 0.01; ****, *P* < 0.0001; dpo, days post-onset of symptoms. PRNT was conducted as reported recently by using Vero cells and testing sera at 1:20, 1:40, 1:80, 1:1,160, 1:320, and 1:640 dilutions (25). Diluted sera were incubated with 50 PFU of either CHIKV or MAYV for 1 h at 37°C before inoculation of cells in 12-well plates. Following inoculation, a carboxymethyl cellulose-Dulbecco's modified Eagle medium (DMEM, containing 2% fetal calf serum) overlay was added. Cells were fixed at 3 days (CHIKV strain 889) or 4 days (MAYV strain TRVL15537) after infection using formaldehyde. Titers >1:5 that reduced the number of PFU by >50% were considered positive. The dashed horizontal line indicates the 1:5 serum dilution cut-off. (B) Median absolute differences between CHIKV and MAYV ELISA reactivities and PRNT titers in longitudinally sampled CHIKV-specific sera. (C) Relative differences between CHIKV and MAYV PRNT titers. The formula for calculation was as follows: relative difference = (higher PRNT titer – lower PRNT titer)/higher PRNT titer. (D) CHIKV and MAYV endpoint PRNT titers in Brazilian CHIKV- and Peruvian MAYV-specific sera. For calculation of endpoint titers and statistical significance, see the legend for panel A. (E) Differences between CHIKV and MAYV IgM ELISA reactivities. (F) Differences between CHIKV and MAYV IgG ELISA reactivities. Positive ratio differences in panels E and F indicate CHIKV infection, and negative differences indicate MAYV infection.

Plaque reduction neutralization testing (PRNT) is the gold standard for arbovirus serology. Testing of longitudinal CHIKV samples showed steadily increasing CHIKV-specific PRNT titers, peaking at 90 dpo. Cross-neutralization of MAYV was rare and occurred more often with increasing CHIKV-specific PRNT titers (Fig. 2A). To compare the levels of robustness of ELISA and PRNT in differentiating between CHIKV and MAYV infection over time, absolute differences between CHIKV and MAYV ELISA reactivities and PRNT titers were calculated for longitudinal CHIKV samples. Differences between CHIKV and MAYV were highest at 5 to 9 dpo for IgM, at 10 to 14 dpo for IgG, and at 90 dpo with PRNT, highlighting the utility of IgM detection in acute infections, IgG detection in early convalescence, and PRNT in late convalescence (Fig. 2B).

In this study, 20% of all PRNT-positive samples showed cross-neutralization. However, comparing PRNT titers allowed unambiguous classification of all samples (Fig. 2C). Mean CHIKV-specific and MAYV-specific PRNT titers differed 40-fold for CHIKV samples and 35-fold for MAYV samples (*P* < 0.0001, Mann-Whitney test) (Fig. 2D).

Parallel PRNT testing for CHIKV and MAYV increased the assays’ positive predictive values (PPV) from 80.0% to 100% (*P* = 0.0053, Fisher’s exact test). We thus compared ELISA reactivities of all samples to examine whether parallel ELISA testing allows differentiation of CHIKV-specific and MAYV-specific antibodies. Assuming relatively higher ELISA reactivity to represent the correct result and lower reactivity to represent a cross-reactive test result, parallel testing significantly increased PPV for IgM detection from 35.3% to 88.2% (Fig. 2E) and for IgG detection from 61.3% to 96.8% (*P* < 0.0001, Fisher’s exact test) (Fig. 2F).

## 

### Discussion.

Consistently with preliminary studies which did not focus on the differentiation of individual viruses (9, 13), we show that the differentiation of CHIKV- and MAYV-specific antibodies based on a single ELISA testing is challenging in regions of cocirculation. Particularly, IgM antibodies were highly cross-reactive, highlighting that false-positive results must be expected during patient diagnostics. Interestingly, IgG cross-reactivity was more frequent among MAYV-specific than among CHIKV-specific sera, which is different from the results of studies describing CHIKV-specific antibodies to be more cross-reactive than o’nyong-nyong virus (ONNV, an alphavirus that is genetically closely related to CHIKV)-specific antibodies. Cross-reactivities of antibodies thus require experimental assessments and cannot be foretold (7). Our results confirmed preliminary data indicating that PRNT is not immune to cross-reactivity but that comparing CHIKV- and MAYV-specific PRNT titers provides robust results, allowing differentiation of both (14). Notably, unambiguous ELISA and PRNT interpretations may be difficult if a person was recently infected by both viruses, a scenario that we could not investigate in our study. However, to what extent potential superinfection exclusion affects CHIKV, MAYV, and other alphaviruses is unclear. Recently, convalescent-phase sera from CHIKV-infected patients were found to cross-neutralize MAYV and the antigenically related Una virus at low titers (14). There is evidence that preexisting CHIKV immunity can also cross-protect from other alphaviruses, including ONNV and the antigenically distant Venezuelan equine encephalitis virus (VEEV) (15–17).

To overcome the low specificity of ELISA and disadvantages of PRNT, including high workloads, new serologic tests allowing the differentiation of CHIKV- and MAYV-specific antibodies are urgently needed (7). Structural protein microarrays (18), in-house ELISAs (19, 20), and competitive ELISAs, such as those developed before for other alphaviruses (21, 22), may provide solutions. However, those methods need to be extensively validated before application in routine diagnostics. Parallel ELISAs for both CHIKV and MAYV provide a convenient and robust solution to ensure specific diagnostics and differentiate CHIKV from MAYV infections. Naturally, this approach relies on the usage of comparable antigens at comparable amounts and comparable test protocols, and thus our results cannot be extrapolated to the usage of any given test before validation.

Our study is limited by the absence of samples from areas where CHIKV and MAYV are coendemic and from PCR-confirmed MAYV infections. However, the robustness of our sample classifications is supported by three arguments: (i) about half of the MAYV samples were collected 10 years before CHIKV arrived in the Americas (Table 1), (ii) classifications are consistent with serologic test results, and (iii) Peru is a hot spot of MAYV circulation, while CHIKV activity is extremely low (2, 23).

Our study provides a template that is amenable for usage in public laboratories dealing with large numbers of samples in resource-limited settings in all regions of alphavirus coemergence, such as CHIKV and ONNV in Africa, CHIKV and Sindbis virus in Asia, equine encephalitis viruses in North and Central America, and Ross River virus and Barmah Forest virus in Australia (24).
